# Calculating the prevalence of soil-transmitted helminth infection through pooling of stool samples: Choosing and optimizing the pooling strategy

**DOI:** 10.1371/journal.pntd.0007196

**Published:** 2019-03-21

**Authors:** James E. Truscott, Julia C. Dunn, Marina Papaiakovou, Fabian Schaer, Marleen Werkman, D. Timothy J. Littlewood, Judd L. Walson, Roy M. Anderson

**Affiliations:** 1 The DeWorm3 Project, Department of Life Sciences, Natural History Museum, London, United Kingdom; 2 Department of Infectious Disease Epidemiology, Faculty of Medicine, Imperial College London, London, United Kingdom; 3 Parasites & Vectors, Department of Life Sciences, Natural History Museum, London, United Kingdom; 4 Department of Global Health, University of Washington, Seattle, Washington, United States of America; Consejo Nacional de Investigaciones Cientificas y Tecnicas, Fundación Mundo Sano, ARGENTINA

## Abstract

Prevalence is a common epidemiological measure for assessing soil-transmitted helminth burden and forms the basis for much public-health decision-making. Standard diagnostic techniques are based on egg detection in stool samples through microscopy and these techniques are known to have poor sensitivity for individuals with low infection intensity, leading to poor sensitivity in low prevalence populations. PCR diagnostic techniques offer very high sensitivities even at low prevalence, but at a greater cost for each diagnostic test in terms of equipment needed and technician time and training. Pooling of samples can allow prevalence to be estimated while minimizing the number of tests performed. We develop a model of the relative cost of pooling to estimate prevalence, compared to the direct approach of testing all samples individually. Analysis shows how expected relative cost depends on both the underlying prevalence in the population and the size of the pools constructed. A critical prevalence level (approx. 31%) above which pooling is never cost effective, independent of pool size. When no prevalence information is available, there is no basis on which to choose between pooling and testing all samples individually. We recast our model of relative cost in a Bayesian framework in order to investigate how prior information about prevalence in a given population can be used to inform the decision to choose either pooling or full testing. Results suggest that if prevalence is below 10%, a relatively small exploratory prevalence survey (10–15 samples) can be sufficient to give a high degree of certainty that pooling may be relatively cost effective.

## Author summary

Current diagnostic methods for assessing prevalence of soil-transmitted helminths (STHs) largely rely on microscopic visualization of helminth eggs, an inexpensive but insensitive method of detection. However, growing interest in going beyond control to break transmission of STH through mass drug administration will require highly sensitive assays to detect the low intensity infections that occur when prevalence is low within a population. Molecular tools, such as real-time PCR, offer the required sensitivity, but depend on well-equipped laboratories and adequately trained technicians. In addition, current assays are relatively expensive to perform at the scale required for surveys. Sample pooling is a technique that can be used to estimate prevalence from a set of samples, while potentially employing fewer tests for a given sample size, reducing cost. The decision in favour of or against pooling will determine how samples are collected, properly stored and analysed, and that needs to be established early in the study or program design process. Our work identifies the key determinants on which this decision should be made, what information is needed to make the choice and how the decision can be made.

## Introduction

Prevalence is a key epidemiological measure for assessing the burden of soil-transmitted helminths (STH) in human communities. The majority of data collected within national surveillance and treatment programs are in the form of prevalence, although infection intensity data based on egg counts from faecal samples can also be collected by using a variety of diagnostic techniques [1]. Treatment guidelines from the World Health Organisation (WHO) for control of STH species use prevalence data to categorise areas into high, medium and low risk and concomitantly define mass drug administration (MDA) treatment strategies accordingly [2]. In the context of public health decision making, intensity data are chiefly used to calculate the prevalence of individuals with low, medium and high intensity infections. Such an approach has been used as a proxy measure for STH infection-induced morbidity in populations [3]. In recent years, interest has started to turn from control of morbidity to the possibility of the elimination of STH infection through breaking the parasite’s transmission cycle in the human population [4,5]. Breaking transmission is achieved by driving infection prevalence through a critical threshold below which parasite reproduction cannot sustain transmission [6,7]. In this context, the ability to accurately measure a very low prevalence will become increasingly important as programmes target elimination.

A variety of diagnostic methods are currently used in assessing prevalence in endemic areas, but most are based on microscopic examination of a standard quantity of stool and the subsequent calculation of the number of eggs it contains [8]. The most frequently used egg-counting method for STH diagnosis is the Kato-Katz (KK) technique [9]. Egg counting methods generally require minimal equipment, beyond a microscope, and testers can acquire the necessary skills within a week of training [10]. However, the sensitivity of these techniques is known to be poor under most conditions, although this is hard to quantify in the absence of a gold standard. This is largely a consequence of heterogeneity in the distribution of egg output in stool excreted by an infected individual with a given worm burden [11,12]. A survey of the literature suggests that the sensitivity of egg counting methods is highly inconsistent across studies. Sensitivity appears to vary according to parasite species, chosen diagnostic method, underlying prevalence/intensity of disease and even across different studies (presumably indicating the importance of the degree of training of the operator/tester). Some patterns emerge however; the test-sensitivity to detect *Ascaris lumbricoides* eggs appears generally high across all background intensities (sensitivity approx. 0.7) [8,13–15] while hookworm-larvae detection in stool is much less reliable, showing a tendency to drop from higher values at high prevalence (sensitivity: 0.65 at 80% prevalence)[15] to low values at very low prevalence (sensitivity: 0.3 at 18% prevalence) [14].

Molecular methods, such as Polymerase Chain Reaction (PCR) and quantitative PCR (qPCR) techniques, offer a significantly more sensitive diagnostic method by detecting STH DNA in the stool specimen, almost certainly from eggs, and amplifying it to ensure more reliable measures of concentration [14,16,17]. The initial quantity of DNA in the sample can be quantified indirectly by counting the number of cycles of amplification necessary to reach a predetermined detection threshold [16]. Sensitivities in the range of 98% are reported, depending on the efficiency of the protocol used for extraction of the DNA [14]. The main drawbacks for these techniques are the expense, the potential for contamination within the laboratory, the need for relatively advanced equipment with a reliable supply chain, and extensive training of laboratory staff. In most cases, field-collected samples need to be properly preserved and stored before they get transported to centralised facilities for subsequent processing and testing [18]. In this situation, pooling of stool samples represents an appealing approach whereby the time and cost (translated to labour time needed to perform a number of DNA extractions and PCR runs) can be significantly reduced while still maintaining the sensitivity of the diagnostic test performed and hence ascertaining the presence of STH species in all the samples collected.

Pooling consists of combining equal portions from each sample that will make up the pool and combining them into one sample, usually with a homogenisation step to ensure sufficient mixing. The number of samples that make up a pool is a key variable of the protocol. Aggregating samples into a pool allows bulk properties of the constituent samples to be measured with a single test. There is a range of theoretical and experimental work on the estimation of faecal egg count by pooling, for STH infections in both animals and humans [19–21]. Comparisons of mean egg counts and egg count reduction rates from individual and pooled samples show reasonable levels of agreement, improving as pool sizes increase [22].

The most common approach is the use of pooling to detect presence of an infectious agent. Assuming sufficient sensitivity, a negative test for the pool implies all constituent samples are negative. The larger the pool size, the fewer tests that need to be made. The process of pooling naturally ‘averages’ the intensities of constituent samples and hence the sensitivity and specificity for detection at pool level may differ from that at the individual sample level. At low levels of infection, a pool consisting of a single positive sample will require greater sensitivity due to an effective dilution. The impact of pooling on the sensitivity of detection and egg count estimation has been investigated using the Kato-Katz diagnostic for STHs and schistosomiasis [23]. The results indicate that the detection sensitivity was considerably reduced by pooling, as expected, while estimated egg counts were generally increased. A similar study based on the POC-CCA diagnostic technique for schistosomiasis found much lower drops in sensitivity with pooling, perhaps reflecting the better inherent sensitivity of the method [24]. Other work has attempted to use detailed models of worm distribution and diagnostics [12] to address sensitivity and specificity for pooled samples for *Schistosoma mansoni* [25,26]. The relationship between the probability of individual samples being positive, and pools being positive, can be used to estimate prevalence from pooled results, but standard estimators can be strongly biased [27].

An extension of the pooling technique is to use it as a method to identify the test status of all samples. In this case, pools with a positive test result have their constituent samples individually tested to identify infected individuals [28]. In this paper, we examine using this approach to calculate the prevalence of STH infection in a population. We also assess the optimal design for a pooling scheme, in order to minimize the number of tests performed.

The main aim of this study is to examine the circumstances under which pooling of samples from a survey to measure prevalence is potentially more cost-effective than testing all samples, where the cost of a survey design is a function of the number of tests or DNA extractions that are required. The question arises specifically in the context of the DeWorm3 project, a cluster randomized trial to test the feasibility of interrupting STH transmission using biannual MDA targeting all age groups funded by the Bill and Melinda Gates Foundation [4,29]. The project includes cross-sectional prevalence surveys conducted using qPCR, to be completed at baseline, mid-point and end-point of the trial. However, without prior information on the prevalence of infection within a cluster, it is impossible to say whether pooling will be more or less cost-effective than testing all samples individually, or indeed what sampling and pooling strategy might be most efficient.

We construct probability distributions for the cost of testing and overall cost-effectiveness of pooling and use both to investigate the dependence of cost effectiveness on the underlying prevalence of STH infection in a given population and the number of samples aggregated into a pool. Note that the method is designed to be used for all stool sample diagnostics for faecal egg detection released by human helminths, not just STHs. We also examine the effect of uncertainty in the underlying prevalence on cost-effectiveness and look at how sparse prior information on prevalence can be used to make decisions on the sampling protocol to be employed.

## Methods

### Expected relative cost of a pooling strategy

Our method for using pooling to calculate prevalence is analogous to that outlined by Corstjens et al for identifying infected individuals through pooling [28]. Samples are grouped in pools of size n. Aliquots from each pool are then extracted and tested using qPCR. Assuming 100% sensitivity for the test, a negative result indicates that all constituent samples are also negative. For pools that test positive, all constituent samples are individually processed and tested to ascertain how many are positive.

For a pool size of n and an underlying prevalence, *π*, the probability of a positive pool is

$$p_{+}=1-{\left(1-\pi \right)}^{n}$$ (1)

Assuming a total number of samples *N*, the number of positive pools, *N*_*+*_, will have a positive binomial distribution

$$N_{+}∼\text{Binom}\left(N_{T},p_{+}\right)$$ (2)

where $N_{T}=N/n$ is the total number of pools. This expression assumes that the number of samples is exactly divisible by the pool size, which would be expected to be a feature of sampling design. The number of tests, *T*, required to calculate prevalence is also a random variable

$$T=N_{T}+nN_{+}$$ (3)

The cost of the testing strategy will be a function of the number of tests performed. Other factors that determine cost include equipment overheads, staff time and the details of the testing protocol as the number of tests needed increases [18]. While such detailed cost data are often not available, they can be acquired. We discuss this important aspect of cost-effectiveness calculation in detail in the Discussion section. Here, we consider the simplest possible case in which there is a fixed cost per test, independent of the number of tests being carried out. With this assumption, we also ignore the time and cost of preparing the pool. One of our key interests is whether a pooling strategy leads to a greater or lesser cost than simply testing all samples. As such, the relative cost, *C*,

$$C=\frac{T}{N}$$ (4)

is useful. If *C* > 1, pooling is not cost-effective in comparison to testing all samples separately, while if *C* < 1, it is more cost effective than the individual testing of all samples. The value of *C* will vary according to how the individual pools are constituted from the samples, so we present results in terms of its mean value and the shape of its distribution. We first derive some key properties of the mean relative cost $\bar{C}$ and how it depends on pool size and underlying prevalence, which provides key insights into the conditions under which pooling can be advantageous.

### Decision criteria for pooling strategies

The mean relative cost is only a single statistic of relative cost. The criterion for deciding whether to proceed with a pooling approach will depend on the details of the relative cost distribution. A better basis for decision-making is the probability that relative cost is less than 1, *P*(*C* < 1). A threshold, *P*_*T*_, can then be defined as the probability that pooling is more cost-effective than testing individual samples. If the condition *P*(*C* < 1)≥ 1−*P*_*T*_ is met, then pooling will be chosen over testing all samples.

Without some information on the prevalence of infection in the population, it is impossible to know whether pooling of any sort will be relatively cost-effective. Some information is required to make such a decision. Information on prevalence might come from limited exploratory testing of the population using standard egg-counting diagnostic methods such as Kato-Katz or molecular techniques. Additionally, there may be prior information from previous studies in the literature, data from national surveys or expert opinion based on local history or conditions.

To combine these ideas, we create a Bayesian model combining prior information about prevalence and exploratory testing data from individual samples to generate the resulting probability distribution for relative cost (See (SI) for derivation and analysis). We define the new relative cost random variable as *C*_*U*_ to distinguish it from *C*, the relative cost when prevalence is known. Details of the derivation can be found in the SI, but the resultant expression for the probability of pooling cost effectiveness is given by

$$P(C_{U}<1)=\sum_{N_{+}=0}^{N_{+}^{C}}P(N_{+}|d_{+},d_{T},N_{T})$$ (5)

Here, $N_{+}^{C}=(n-1)N_{T}/n$ is the sample size, expressed in terms of pool test results. The conditional probability is

$$P(N_{+}|d_{+},d_{T},N_{T})\propto \int_{\pi =0}^{1}P(N_{+}|\pi ,N_{T})P(d_{+}|d_{T},\pi )P(\pi )d\pi$$ (6)

where the first probability under the integral is the binomial probability of *N*_*+*_ positive pools, the second is the probability of *d*_*+*_ positive exploratory tests out of *d*_*T*_ trials with prevalence, *π*, and the last term, *P*(*π*), represents other prior information about prevalence. The derivation of this relationship can be found in the SI. We use this model to examine the dependence of the probability of efficient pooling, and hence the decision to pool, on the amount and type of information available on the underlying prevalence of disease.

## Results

We first consider the case in which the underlying prevalence of infection, *π*, is known. The value of the relative cost, *C*, can vary in the range [1/*n*,1+1/*n*], where pooling is cost effective when *C* < 1. The distribution of relative cost is examined further below and in the SI, but the dependence of relative cost on pool size and prevalence can be seen by considering the case in which the expected relative cost, $\bar{C}=1$. This acts as a discriminant, dividing cases in which the expected cost effectiveness is positive for pooling from those in which it is negative. In the SI, we calculate the prevalence as a function of pool size for unit relative cost as

$$\pi_{c}(n)=1-\text{exp}\left\{-\frac{\text{ln}(n)}{n}\right\}$$ (7)

**Fig 1:**
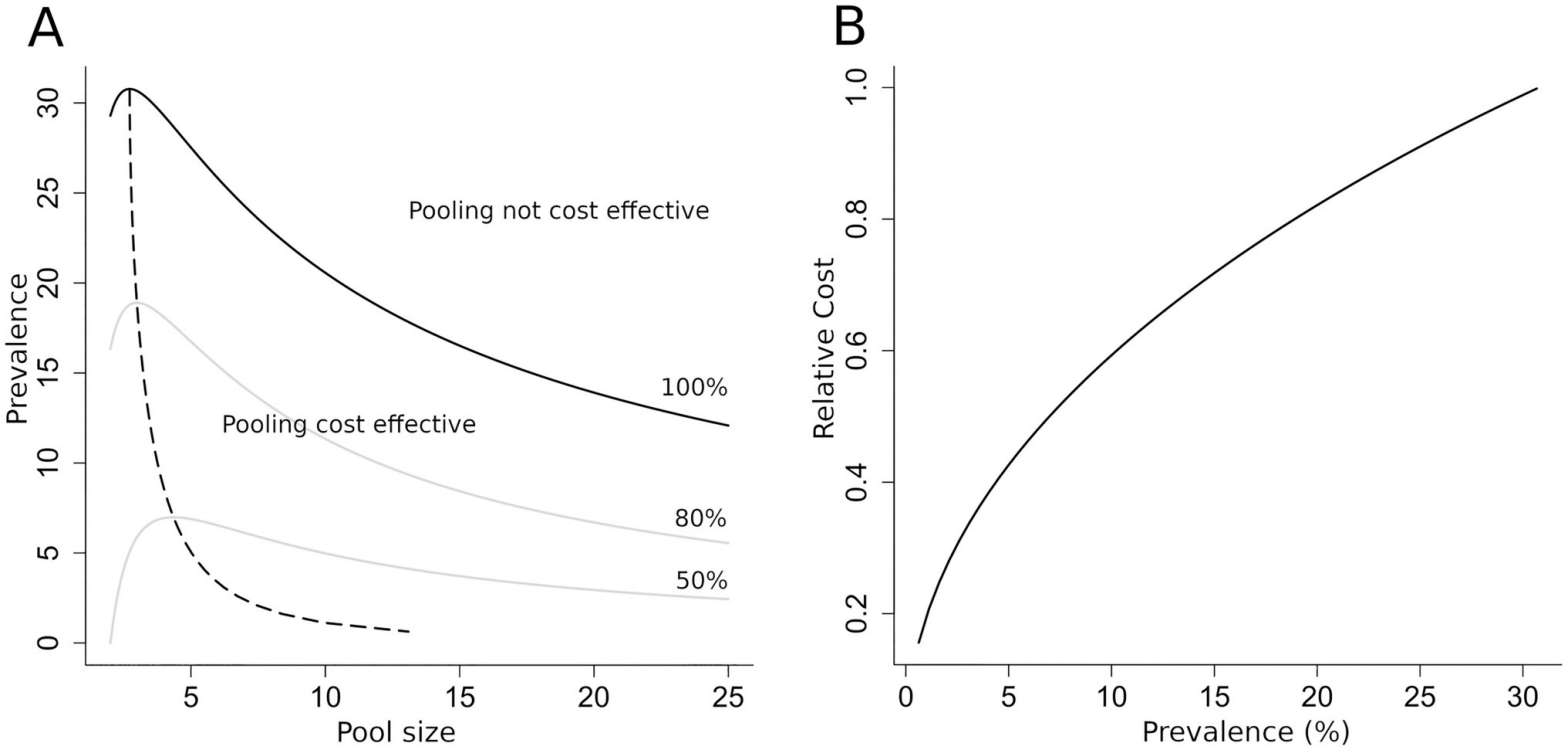
Expected relative cost as a function of prevalence and pool size. A) Combinations of prevalence and pool size for which pooling is cost effective or not, on average. Solid line indicates combinations for which pooling and individual sampling are matched. Grey lines indicate contours for relative cost at 50% and 80% (as formulated by equation S5 in the SI). Dotted line shows most cost-effective pool size for a given prevalence. B) Relative cost of optimum pool size as a function of prevalence. Pool sizes for prevalence are given by the dotted line in panel A.

This discriminant characterises any combination of pool size and prevalence as, on average, cost effective or not (Fig 1). For a given pool size, prevalence below *π*_*c*_(*n*)are cost effective, on average. In the figure, pool size is an integer quantity and the continuous line of the discriminant in the figure is a series of points at whole number values. Critical prevalence has a maximum value

$$\pi_{c}^{\text{max}}=1-\text{exp}\left\{-\frac{1}{e}\right\}≃0.31$$ (8)

Consequently, pooling is, on average, not cost effective for all prevalence greater than 31%, independent of the size of the pools. The maximum critical prevalence is achieved for *n* = *e* ≃ 2.72. Hence the pool size which is cost effective for the largest range of prevalence is 3, the nearest integer. As pool size increases, the critical prevalence for cost effectiveness falls.

Within the cost-effective region, the optimally pool size (on average) for a given prevalence is shown by the dotted line in Fig 1A. Optimal pool size increases slowly with decreasing prevalence but doesn’t rise above 5 until prevalence is below 5%. Below this prevalence, however, optimal pool size grows rapidly with decreasing prevalence. Fig 1B shows how the mean relative cost effectiveness for optimum pool size varies with prevalence. As prevalence decreases below the value at which pooling becomes a viable option, the cost effectiveness gradually increases. For prevalence values around 2%, pooling on average requires only a fifth of the number of extractions and tests as would be required if testing samples individually.

The effect of uncertainty in the underlying infection prevalence is analysed in detail in the SI. We present the main results in this section. The introduction of uncertainty in underlying prevalence has two effects on the probability distribution of relative cost. Firstly, uncertainty of the underlying prevalence increases the overall variance. The variance including uncertainty is (See SI)

$$\text{var}(C_{U})=\frac{N_{T}-1}{N_{T}}\text{var}(p_{+})+\text{var}(C)$$ (9)

where *C*_*U*_ is the relative cost with uncertainty in the underlying prevalence. The variance is the sum of the variance associated with pool construction and that associated with the uncertainty in the underlying prevalence. For any reasonable number of pools employed, the first term is effectively var(*p*_+_). The mean cost effectiveness is also altered by the additional uncertainty in prevalence. The mean can be expressed as

$${\bar{C}}_{U}=\frac{1}{n}\text{+}1-\frac{B(d_{+}+1,d_{T}-\bar{\pi }d_{T}+n+1)}{B(d_{+}+1,d_{T}-\bar{\pi }d_{T}+1)}$$ (10)

where *B*(…) is the beta function, *d*_*T*_ is the total number of exploratory tests and $\bar{\pi }=d_{+}/d_{T}$ is the expected underlying prevalence. In general, it is not clear whether ${\bar{C}}_{U}$ is greater than or less than $\bar{C}$; that is, whether the uncertainty in prevalence leads to a higher or lower relative cost.

**Fig 2:**
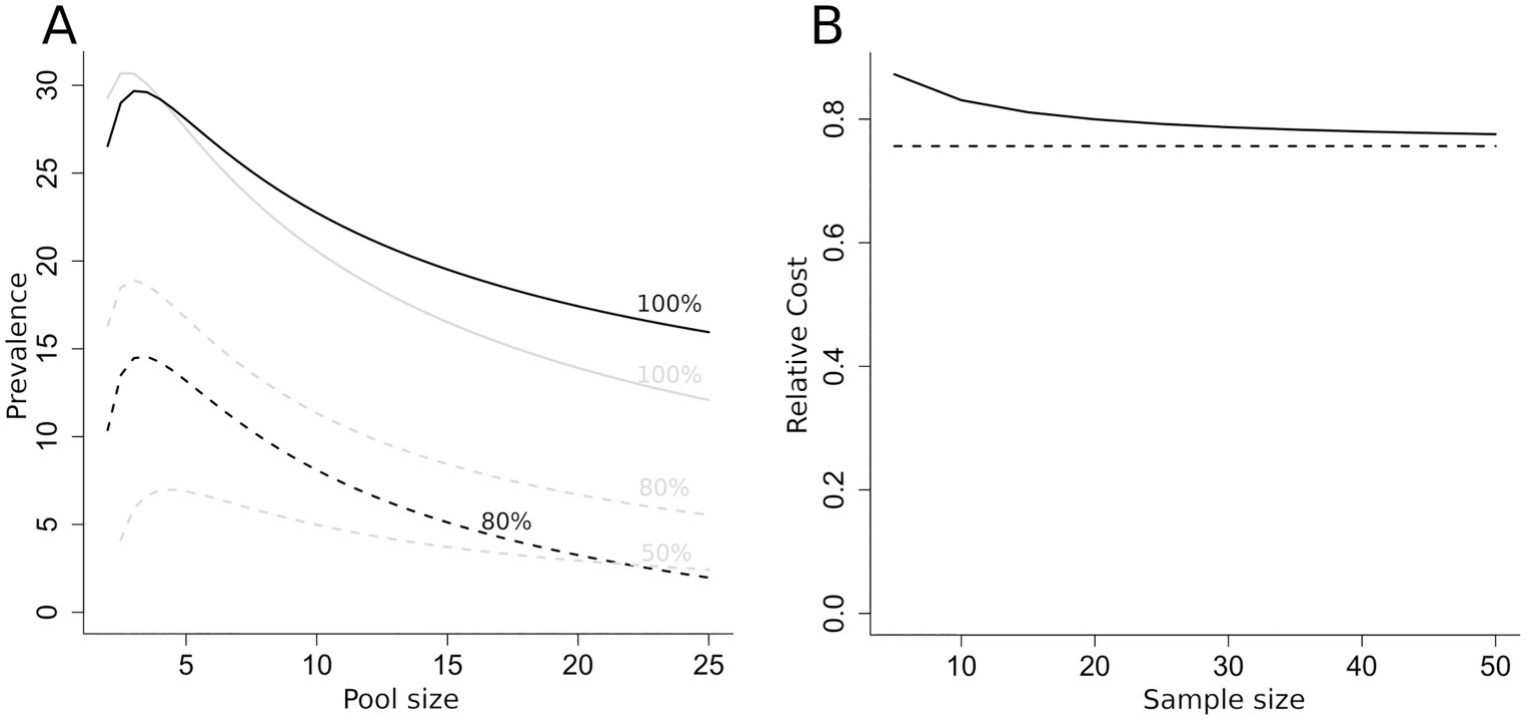
The effect of prevalence uncertainty on mean relative cost. A) Prevalence for expected relative cost of 100%, 80% and 50% with sample size of 10. Grey lines show relative costs for known prevalence, as in Fig 1A. Y axis indicates known underlying prevalence or mean prevalence from exploratory data when prevalence is unknown. B) Relative cost against exploratory sample size. Broken line shows relative cost when prevalence is known and set to the mean from the exploratory tests. (Mean prevalence 15%, pool size of 5).

However, it is the case that for low prevalence situations where $\bar{\pi }<1/(n+1)$, the cost is increased by uncertainty (See SI). Fig 2 shows the effect of uncertainty in prevalence on relative cost.

Fig 2A shows the effect on the critical prevalence/pool size relationship for *d*_*T*_ = 10, effectively correcting Fig 1A for the effects of uncertainty in underlying prevalence. Critical prevalence (100%) is higher for larger pool sizes but lower for the most cost-effective lower pool sizes, but the effect is not large. The maximum prevalence for cost effective pooling is only marginally affected. The figure also compares contours for 80% and 50% relative cost between known and uncertain prevalence. Prevalence uncertainty increases the separation of the 100% and 80% cost contours, with 80% achievable only below a prevalence of 15%. A relative cost of 50% is not possible within the range of pool sizes examined. As shown in Fig 2B, the difference between the uncertain and known prevalence result drops as the sample size increases and the differences become negligible for sample sizes above 50.

**Fig 3:**
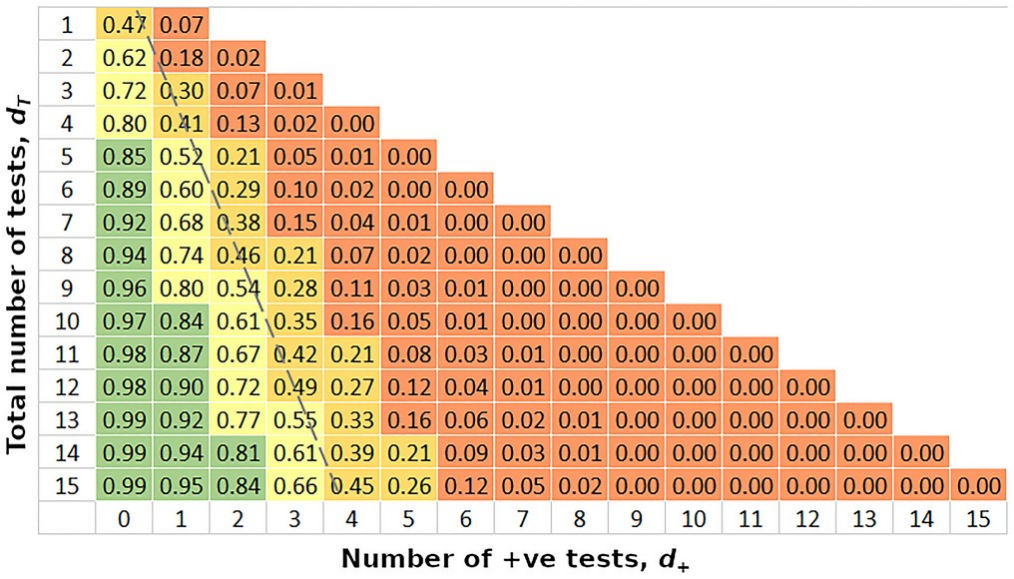
Probability of greater efficiency using pooling, as a function of the number of exploratory tests and the number of positive results. The exploratory diagnostic test is assumed to be perfect in this case. Prior knowledge of prevalence is uninformative. Pool size = 5. Green => 80%, yellow = 80% -> 50%, orange = 50% -> 20%, red <= 20%. The broken grey line approximately tracks the critical prevalence for 100% relative cost for pool size of 5 and exploratory sample size of 10.

Fig 3 shows the probability that pooling will be cost effective, given the number of exploratory tests (on the left) and the number of those that are positive (along the bottom). The prevalence prior is set to be uninformative. The diagnostic test in the exploratory phase is assumed to have 100% sensitivity and specificity in this case and pool size is set at 5. Taking the probability of cost effectiveness, *P*_*T*_ = 80% as the pooling choice threshold, the green region of Fig 3 defines the exploratory results that would trigger the use of pooling for this size of pool. Five or more negative tests with no positives exceeds the threshold, as does 10 or more tests with one or fewer positives. Having reached the threshold for choosing pooling, it’s possible that further information can invalidate that choice. For example, 5 negative tests suggest pooling, but if the next test were positive, the support for pooling would drop to 60%, and as such below the chosen threshold. The band of yellow and orange result combinations that run diagonally across the figure correspond to the critical prevalence for pool size of 5. Fig 2A shows this to be about 27% for 10 samples. The broken grey line tracks this prevalence across the table, approximately.

**Fig 4:**
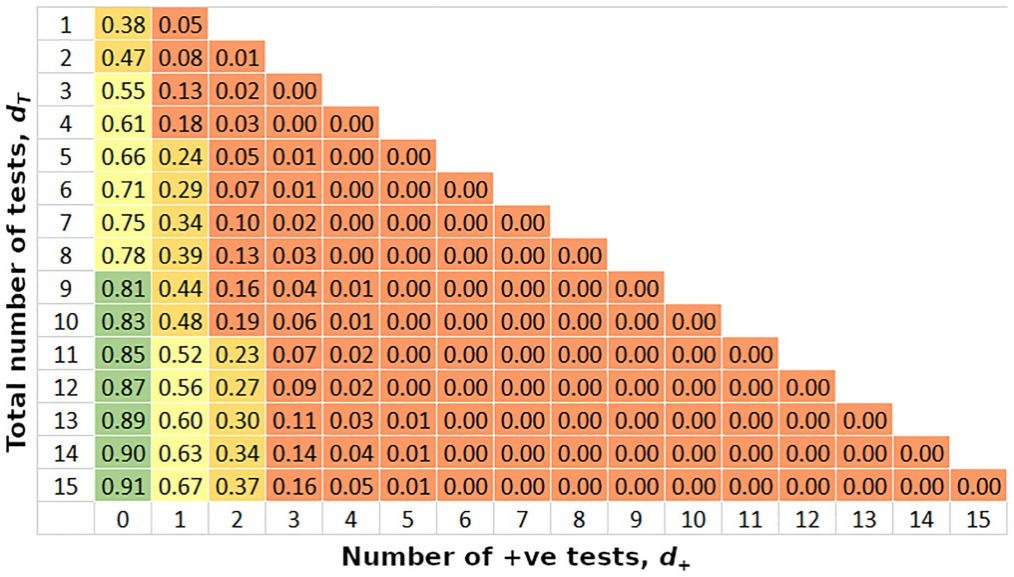
Probability of greater efficiency using pooling, as a function of the number of exploratory tests and the number of positive results. The exploratory diagnostic test is assumed to have a sensitivity that varies smoothly from 30% for the lowest prevalence to 70% for the highest. Prior knowledge of prevalence is uninformative. Pool size = 5. Green => 80%, yellow = 80% -> 50%, orange = 50% -> 20%, red <= 20%.

Kato-Katz sensitivities for hookworm vary considerably (25–65%) and appear to decline as prevalence reduces in a roughly linear fashion (evidence from the literature on this is very variable and often hard to interpret). Our models of diagnostic sensitivity as a function of prevalence indicate that for hookworm, sensitivity is increasing approximately linearly with underlying prevalence. Fig 4 shows the support for pooling for hookworm with sensitivity varying from 30% at the lowest prevalences to 70% for 100% prevalence. This increases the number of samples needed to support a decision to pool, but does not change the basic structure i.e. support for pooling is only generated by finding very low numbers of positive samples.

## Discussion

Analysis of mean relative cost reveals some of the main qualitative and quantitative features of pooling as compared to complete processing of individual samples. The line defining neutral relative cost, $\bar{C}=1$, suggests that the maximum prevalence for which pooling could be advantageous is given by $\pi_{c}^{\text{max}}$, which is approximately 31%. The closest integer pool size to this point of maximum effectiveness is at a pool size of three (Fig 1A). The value of the maximum prevalence and the pool size at which it occurs do not vary significantly with uncertainty as to the underlying prevalence.

Analysis shows that the most efficient pool size for prevalence values below $\pi_{c}^{\text{max}}$ remains below five down to approximately 5% prevalence. However, below 5%, there is a rapid increase in optimal pool size, with a 2% prevalence having an optimal pool size close to 10. It should be noted that pool sizes of five are considerably smaller than those used purely for the detection of the presence of infectious agents, rather than assessing the prevalence of infection [19]. The relative cost for optimal pool size is shown in Fig 1B. The trend indicates that the relative cost is minimum for the lowest prevalence values. A prevalence rate below about 10% indicates that pooling is less than half the cost of testing all samples individually.

The shape of the optimal pool size curve in Fig 1A suggests that, in the absence of accurate prevalence information, 5 is a good default pool size. However, in areas that have been subjected to multiple rounds of MDA, a larger pool size of 10 would lead to more cost effective pooling, assuming no loss of sensitivity. In particular, this would apply in the case of transmission assessment surveys (TAS). Currently, there is no formally endorsed TAS for STHs, but they typically involve large sample sizes to compare measured prevalence to threshold values at the 1–2% level [30]. Key to the use of pooling in this context, however, would be assessing whether pooling sensitivity could still reliably be assumed to be 100% for pooled samples from lightly infected individuals.

The issue of qPCR sensitivity to low egg counts and, by extension, sample dilution, is made complicated by the nature of the qPCR process. A positive result for a sample is the result of a detection threshold being reached within a certain number of amplification cycles (the CT value). Detection sensitivity as a function of egg count or dilution therefore tends to have a binary character. If the threshold is attained within the maximum number of cycles used, the sensitivity tends to be close to 100%. When CT values get close to the maximum value, sensitivity drops rapidly to very low levels. Additionally, the detection threshold and the maximum number of cycles performed are calibrated with respect to the preparation protocol to be used for a batch of samples. As a result, point at which sensitivity falls away is very dependent on the particular experimental set up and hard to generalise. The characteristics of sensitivity as a function of dilution are the subject of current research.

The introduction of uncertainty in the underlying prevalence both increases the variance of the relative cost and also biases its mean value. However, as shown in Fig 2, even with quite poor information on prevalence, the bias in expected relative cost is only plus or minus a few percentage points. Relatively little information on prevalence is required to achieve good support for a pooling approach. If the exploratory data is assumed to have 100% sensitivity, as would be the case for qPCR, as few as 5 individual tests can be sufficient to reach 80% support for pooling if the underlying prevalence is very low (Fig 3). Support of 80% for individual sampling can be achieved with only a couple of data points, if the underlying prevalence is very high. There is a range of exploratory data results that give no clear indication for either strategy (that is, support for pooling is between 20% and 80%). This occurs when exploratory results indicate prevalence is close to the critical value at which relative cost is 1. The decision for or against pooling will be most difficult when the costs of the two alternatives are closely balanced.

When exploratory data are collected through Kato-Katz diagnostics, the reduced sensitivity increases the amount of data necessary for a decision to be made (Fig 4). This situation is likely to be quite common, as most prevalence surveys are currently conducted using microscope-based techniques. However, in order to take advantage of this, the sensitivity of the Kato-Katz method would have to be well characterised. Since this feature of KK is so poorly documented and is context- and technician-dependent, it would be necessary to use the most pessimistic sensitivity figures, which would push up the amount of data required for a decision and biasing the decision process in the direction of caution and against pooling. However, the situation seems very different for the highly sensitive PCR diagnostic method; the method further benefits from inclusion of internal controls to ensure successful extraction of the DNA/target present and from the consistency of application with accepted ranges of the detectable value measured assuming a standardized level of technician competency.

In this study, we have used a relative cost metric as the basis on which to discriminate between pooling and testing of all samples separately. Relative cost, as defined in the Methods section, has the advantage that it is simple to interpret and is a linear function of the number of tests performed. The cost metric comprises two parts; the function that converts the number of tests done into a cost (the cost function) and the way in which the cost of pooling and of testing all samples are compared.

The cost of a sampling strategy is made up of the cost of consumables, such as reagents, primers and extraction kits, and the cost of staff time and equipment requirements. For consumables, there is the possibility of economies of scale through bulk buying. These would introduce non-linearities into the relationship between number of extractions and cost, which would apply to both strategies. However, the cost of consumables is likely to be swamped by the much larger costs of machinery purchase and staff salaries for highly-trained technicians. These costs are likely to scale linearly with numbers of tests, since they reflect the number of man-hours per test. Non-linear effects may arise if more equipment or technicians are required to manage the workload. Automated sample processing, extraction and PCR offers the opportunity to significantly reduce the cost of personnel at the expense of larger up-front equipment costs.

The process described as testing in the main text comprises three stages. The most time-consuming stage, and hence the highest cost, is the extraction of DNA from samples, although the pooling process represents a small but not insignificant amount of work [23]. This requires bead-beating and centrifuging of samples [31]. The final PCR amplification of extracted DNA is much less labour intensive, but may be limited by the availability of PCR machines. For pooled samples, there is a preliminary stage of creating pools from individual samples [22]. Hence pooling strategies require some additional work, but it is small in comparison to extraction steps. Such assumptions do require validated approaches using the most efficient pooling approach. The small additional cost associated with pooling samples will reduce slightly the relative cost of pooling described in our analysis. Taking this into account, the neutral cost effectiveness curve of Fig 1A would drop down to slightly lower prevalence values. In order to assess the absolute cost of different sampling strategies, detailed cost information on the different stages of testing will be required. It is likely that this kind of cost information will be context-specific but it should be easy to collect.

The relative cost metric used in the current work has the advantage that absolute costs cancel out, provided the cost functions are linear with the same cost per test. However, this may not be the best cost metric for decision making. It is possible that relative cost may be less important than the absolute difference in outlay between the two sampling approaches. In that case, the difference in cost may be a better metric. While this wouldn’t affect the expected cost-effectiveness as a function of prevalence and pool size as shown in Fig 1, decisions based on exploratory data would now need to be based on the probability of achieving a given cost or saving with respect to testing all samples. This may result in different decisions being made than those suggested by analyses based on relative costs. The theory described in this paper can be easily adapted to address the use of different metrics.

Two potential features of STH prevalence surveys not considered in this paper are the prevalence of the different species that make up the STH classification, and spatial or geographical heterogeneity in infection prevalence. In the case of multiple species, each species will require an independent PCR run for detection if using a singleplex or multi-parallel protocol. However, the pooling and DNA extraction stages of testing will act on all species present simultaneously and these are the costliest parts of the process. Hence the optimal protocol is probably to use pooling for all species of interest if it is indicated as optimal for any of them.

Large-scale surveys are likely to comprise sub-regions across which prevalence will vary. Similarly, cluster-based trials will feature clusters containing a range of prevalence values in sub-populations (or villages) within the cluster. If a wide range of prevalence is indicated (based on prior prevalence information), greater cost effectiveness may be achievable by assigning sub-regions or clusters to different sampling strategies. Whether this is justified will depend on what additional costs are incurred by having a dual sample strategy. However, these costs, if known, could be included in the decision-making analysis.

The analysis presented here represents a basic framework for understanding the cost effectiveness of pooling strategies and a guide to decision-making about the best strategy to adopt in any given setting. Our findings provide a general picture of when to use pooling, how to judge pool sizes, and how prior information can be used to make better decisions. When considering pooling strategy and decisions in specific cases, details of pooling protocols, costs and decision-making priorities will need to be factored into the model and the appropriate calculations made as detailed in this paper. Acquiring detailed cost data is an important requirement in applying these methods.

## Supporting information

S1 File Model derivation and analysis. (PDF)
