# Supplementary material for: Calculating the prevalence of soil-transmitted helminth infection through pooling of stool samples: Choosing and optimizing the pooling strategy
Source: PLoS Negl Trop Dis. 2019 Mar 21;13(3):e0007196. doi: 10.1371/journal.pntd.0007196 (PMC6445468; doi:10.1371/journal.pntd.0007196)
Supplement: S1 File — (PDF) [file pntd.0007196.s001.pdf]

Supplementary information: model derivation and analysis

It is convenient to express the outcome of the pooling process in terms of N_+, N_T , the number of positive pool results and the total number of pool tests. These are related to the total number of tests, *T*, and the total number of samples, *N*, by

$$\begin{aligned} T &= N_T + nN_+ \\ N &= nN_T \end{aligned} \qquad \text{S1}$$

where *n* is the pool size. Relative cost, C, can be written in terms of the number of positive pools

$$C = \frac{T}{N} = \frac{1}{n} + \frac{N_+}{N_T} \qquad \text{S2}$$

The probability of pooling being efficient is then

$$P(C < 1) = P\left(N_+ < \frac{(n-1)}{n} N_T \right) \qquad \text{S3}$$

If the underlying prevalence, *π*, is known, $N_+ \sim Bin(N_T, 1-(1-\pi)^n) = Bin(N_T, 1-p_-)$, where $p_-(\pi) = (1-\pi)^n$. The variance of C is given by $\mathrm{var}(C) = p_-(1-p_-)/N_T$ and the probability expressed in S3 can be written in terms of quantiles of the binomial distribution. The mean of the relative cost, $\bar{C}$, is given by

$$\bar{C}(\pi, n) = \frac{1}{n} + \frac{\bar{N}_+}{N_T} = \frac{1}{n} + 1 - \exp(n \ln(1-\pi)) \qquad \text{S4}$$

For a given value of the relative cost, $\bar{C} = q$, we can rearrange S4 to give prevalence as a function of pool size for a given relative cost.

$$\pi(n; q) = 1 - \exp\left\{ \frac{1}{n} \ln(1 - q + 1/n) \right\} \qquad \text{S5}$$

In the critical case for which the relative cost is 1,

$$\pi(n;1) = 1 - \exp\left\{ -\frac{\ln(n)}{n} \right\} \qquad \text{S6}$$

In the absence of a known prevalence, its value has to be inferred from prior information and data from exploratory sampling. The quantities π and $p_-(\pi)$ become random variables. Let d_+, d_T be the number of positive tests and the total number of tests in the exploratory phase, respectively, and consider the joint probability, $P(N_+, d_+, d_T \mid \nu_T)$. We can write

$$P(N_+, d_+, d_T \mid N_T) = P(N_+ \mid d_+, d_T, N_T) P(d_+, d_T) \qquad \text{S7}$$

The first term on the right is the probability of N_+ positive pools, given a total of N_T and exploratory results, d_+, d_T. This is what we want to calculate. We can also express the joint probability in terms of an augmented joint distribution

$$P(N_+, d_+, d_T \mid N_T) = \int_{\pi=0}^{1} P(N_+, d_+, d_T, \pi \mid N_T) d\pi \quad \text{S8}$$

where π is the unknown prevalence. Sequentially conditioning of the augmented distribution leads to a product of distributions.

$$\begin{aligned} P(N_+, d_+, d_T, \pi \mid N_T) &= P(N_+, d_+ \mid d_T, \pi, N_T) P(d_T, \pi \mid N_T) \\ &= P(N_+, d_+ \mid d_T, \pi, N_T) P(d_T) P(\pi) \\ &= P(N_+ \mid d_+, d_T, \pi, N_T) P(d_+ \mid d_T, \pi, N_T) P(d_T) P(\pi) \\ &= P(N_+ \mid p_-(\pi), N_T) P(d_+ \mid d_T, \pi) P(d_T) P(\pi) \end{aligned} \quad \text{S9}$$

Here, d_T, π, N_T are assumed independent of each other. Equations S7-9 can be combined to give the probability of N_+ positive pool results as

$$P(N_+ \mid d_+, d_T, N_T) = \mathrm{A} \int_{\pi=0}^{1} P(N_+ \mid p_-(\pi), N_T) P(d_+ \mid d_T, \pi) P(\pi) d\pi \quad \text{S10}$$

where A is a normalising factor that can be evaluated by summing across N_+. The first distribution in the integral is the probability of N_+ positive tests out of N_T, given prevalence π, as used in the main text.

$$P(N_+ \mid p_-(\pi), N_T) = \mathrm{Binom}(N_+; N_T, 1 - p_-(\pi)) \quad \text{S11}$$

The second is the probability of d_+ positive exploratory tests out of d_T trials with prevalence, π, which is defined by a beta distribution. The last term, $P(\pi)$, represents other prior information about prevalence. The probability defined in equation S10 can be used to define a probability of pooling efficiency that includes uncertainty in prevalence, C_U.

$$P(C_U < 1) = \sum_{N_+=0}^{N_+^C} P(N_+ \mid d_+, d_T, N_T) \quad \text{S12}$$

where $N_+^C = (n-1) N_T / n$, as described in equation S3.

Some statistics of C_U can be calculated in closed form. For the expected value of C_U,

$$\overline{C}_U = \frac{1}{n} + \frac{1}{N_T} \mathrm{E}[N_+] = \frac{1}{n} + 1 - \overline{p}_- \quad \text{S13}$$

The expectation of $p_-(\pi)$ can be expressed in terms of beta functions

$$\overline{p}_- = \frac{B(d_+ + 1, d_T - d_+ + n + 1)}{B(d_+ + 1, d_T - d_+ + 1)} \quad \text{S14}$$

For comparison, the expected relative cost when underlying prevalence π is equal to the expected value, d_+ / d_T, is given by

$$\bar{C} = \frac{1}{n} + 1 - \left(\frac{d_T - d_+}{d_T}\right)^n \quad \text{S15}$$

The values of $\bar{C}$ and $\bar{C}_U$ are clearly not the same and which is larger depends on the exploratory test data. It is simple to show that for $d_+ > d_T / 2$, $\bar{C} > \bar{C}_U$ and for $d_+ < d_T / (n+1)$, $\bar{C} < \bar{C}_U$ (see Lemma 1, below). Consequently, on average and for low prevalences, the relative cost when there is limited information about prevalence is higher than the cost when prevalence is accurately known. For pool sizes of 4 or more, this falls within the range of prevalence where pooling is more efficient than full testing, $\pi_c^{\max} \simeq 0.31$.

The variance of C_U is given by

$$\mathrm{var}(C_U) = \frac{1}{N_T^2}\mathrm{var}(N_+) = \frac{N_T - 1}{N_T}\mathrm{var}(p_-) + \frac{1}{N_T}\bar{p}_+(1 - \bar{p}_+) = \frac{N_T - 1}{N_T}\mathrm{var}(p_-) + \mathrm{var}(C) \quad \text{S16}$$

The variance comprises two terms. One captures the uncertainty in the composition of the pools and is equal to the variability in C, the relative cost when prevalence is known. The second term arises from the uncertainty in the value of the underlying prevalence. The variance of p_- can also be expressed using beta functions

$$\mathrm{var}(p) = B(d_+ + 1, d_T - d_+ + 2n + 1) / B(d_+ + 1, d_T - d_+ + 1) - \bar{p}_-^2 \quad \text{S17}$$

Lemma S1

i) For $d_+ < d_T / (n+1)$,

$$\bar{p}_- < \left(\frac{d_-}{d_T}\right)^n$$

ii) For $d_+ > d_T / 2$,

$$\bar{p}_- > \left(\frac{d_-}{d_T}\right)^n$$

The probability $\bar{p}_-$ can be written

$$\bar{p}_- = \frac{B(d_+ + 1, d_T - d_+ + n + 1)}{B(d_+ + 1, d_T - d_+ + 1)} = \frac{(d_- + n)}{(d_T + n + 1)}\frac{(d_- + n - 1)}{(d_T + n)}\cdots\frac{(d_- + 1)}{(d_T + 2)}$$

Terms on the right hand side are in descending order of magnitude. For the first term on the right hand side,

$$\frac{d_-}{d_T} > \frac{(d_- + n)}{(d_T + n + 1)}, \text{ if } d_- > \frac{n}{n+1} d_T \text{ or } d_+ > \frac{d_T}{n+1}$$

Hence, all terms on the RHS are less than d_- / d_T and $\overline{p}_- < (d_- / d_T)^n$. Conversely, if $d_+ > d_T / 2$, $\overline{p}_- > (d_- / d_T)^n$.
